# Genomic characterization of *APEC* phages and evaluation of the efficacy in reducing the loads of *APEC* O_78_ infections in chickens

**DOI:** 10.3389/fmicb.2026.1670169

**Published:** 2026-01-29

**Authors:** Qin Lu, Xinxin Jin, Zui Wang, Rongrong Zhang, Yunqing Guo, Qiao Hu, Wenting Zhang, Tengfei Zhang, Qingping Luo

**Affiliations:** 1 Key Laboratory of Prevention and Control Agents for Animal Bacteriosis (Ministry of Agriculture and Rural Affairs), Institute of Animal Husbandry and Veterinary, Hubei Academy of Agricultural Sciences, Wuhan, China; 2 Hubei Provincial Key Laboratory of Animal Pathogenic Microbiology, Institute of Animal Husbandry and Veterinary, Hubei Academy of Agricultural Sciences, Wuhan, China; 3 Hubei Hongshan Laboratory, Wuhan, China

**Keywords:** antibiotic alternatives, *APEC*, chicken, infection, phage

## Abstract

**Introduction:**

The resistance of avian pathogenic *Escherichia coli* (*APEC*) poses a serious challenge to the control of bacterial diseases in the poultry industry. Identification of useful phages as alternatives to antibiotics for *APEC* O_78_ is a priority.

**Methods:**

The phage LQ5 was isolated from the contents of the chicken intestines. Whole-genome sequencing was performed using the Illumina NovaSeq 2500 platform, and then bioinformatics analysis was conducted on the genome. The application effect of LQ5 in the O_78_ infection model of chickens was systematically evaluated.

**Results:**

The phage LQ5 was identified as a member of *Myoviridae* by electron microscopy. Whole-genome sequencing showed that phage LQ5 is a double strand DNA virus with a genome of 171,908 bp containing active components, such as endolysin, holin lysis mediator. Comparison of the bacterial load of *APEC* in chicken liver and spleen tissue in samples treated with phage LQ5 and Amoxicillin showed that the phage LQ5 reduced the bacterial load compared with the antibiotic.

**Discussion:**

These results have enriched the information of the phage gene bank for *APEC*, laying the foundation for the development of targeted phage biocontrol agents against the *APEC* O_78_ strain.

## 1 Introduction

Avian pathogenic *Escherichia coli* (*APEC*) causes colibacillosis, a severe systemic disease in poultry. This disease manifests as airsacculitis, pericarditis, perihepatitis, and septicemia, resulting in substantial global economic losses (de Oliveira et al., 2021; Dziva and Stevens, 2008). *APEC* comprises over 180 serogroups, with serotypes O_1_, O_2_, and O_78_ being the most prevalent and clinically significant in chickens (Halfaoui et al., 2017). An analysis of 189 studies on *APEC* reveals a wide distribution of common O serotypes, with O_78_ being the most prevalent (16%), followed by O_2_ (10%) and O_117_ (8%) (Jhandai et al., 2024).

*APEC* is one of the extraintestinal *E. coli*, which may cause the spread of zoonotic diseases through contaminated eggs (Abdelhamid et al., 2024). The *E. coli* infections in the neonatal poultry are being characterized by septicemia. The pathogenicity of *APEC* is mediated by a diverse arsenal of virulence factors that facilitate colonization, immune evasion, and systemic invasion. Genomic analyses highlight the critical role of adhesins (such as Type-I and P fimbriae), iron acquisition systems (e.g., aerobactin), and secretion systems (notably the Type VI Secretion System, T6SS, which is highly prevalent) in its success (World Health Organization, 2006; Hong et al., 2016). These factors enable *APEC* to infect extra-intestinal tissues, including the lungs, liver, heart, and brain, making it a formidable pathogen.

Current control strategies rely heavily on antibiotics and biosecurity measures. However, the overuse and indiscriminate application of antibiotics in the poultry industry have fueled the emergence of multidrug-resistant *APEC* strains (Naghizadeh et al., 2019). A review of 15 (35.71%) studies about *E. coli* reported 2,269 (35.59%) MDR strains, with 763 (11.97%) strains being ESBLs (extended-spectrum *β*-lactam) and 82 (1.29%) being CREs (carbapenem-resistant Enterobacteriaceae) (Vulcanescu et al., 2024). This practice poses a severe threat to public health, as resistance genes can transfer via mobile genetic elements to zoonotic bacteria, compromising the efficacy of antibiotics used in human medicine. And a study shows the bacteriphage have the greater potential in the alternative management of colistin-resistant *E. coli* infections (Zhu et al., 2024). Thus, more and more bacteriphage need to develop effective alternatives to antibiotics for controlling *APEC* infections. Nevertheless, infections in poultry at veterinary clinical can lead to food safety in the food chain, the main methods of controlling contamination by foodborne pathogens often involve the application of antimicrobial agents, which are now becoming less efficient. There is a growing need for the development of new approaches to combat these pathogens, especially those that harbor antimicrobial resistant and virulent determinants (Oluwarinde et al., 2023).

Bacteriophages (phages), which specifically infect and lyse bacteria, offer a viable therapeutic alternative. The principal advantage of phage therapy lies in its high specificity, enabling it to target pathogenic bacteria while sparing the beneficial host microbiota, which is a frequent limitation of broad-spectrum antibiotics (Qin et al., 2024; Tawakol et al., 2019). Both domestic and international research has confirmed the potential of phages in combating multidrug-resistant *APEC*. Zhang et al. (2025) found that phage YP6 can effectively lyse bacterial strains and inhibit biofilm formation. In a parallel development, a foreign study (Norambuena et al., 2025) demonstrated that AC-01, a cocktail preparation composed of four phages, exhibited lytic activity against over half (56.3%) of the tested bacterial strains. These findings collectively indicate that phage therapy is expected to become an effective approach for preventing and controlling multidrug-resistant *APEC* infections. Many studies have focused on phage therapy against *APEC* in general, but the high serotype diversity demands a targeted approach. Serotype O_78_ remains one of the most dominant and virulent strains circulating globally. However, the development of specifically tailored and highly effective phage cocktails against this key serotype is underexplored. Furthermore, a comprehensive evaluation that combines detailed *in vitro* characterization of phage virulence factors (like host range and kinetics) with robust *in vivo* efficacy data in animal models is essential for translating phage therapy into practical applications.

This study isolated and characterized a novel phage exhibiting potent lytic activity against *APEC* O_78_. We systematically evaluated its efficacy both in vitro and in a chicken challenge model. These findings establish a foundation for developing a targeted, phage-based biocontrol agent against *APEC* O_78_ isolates. This approach presents a potential strategy to mitigate economic losses and reduce reliance on antibiotics in poultry production.

## 2 Materials and methods

### 2.1 Background of bacterial strains

The host bacteria were 10 strains of *E. coli* of serotypes O_1_, O_2_ and O_78_ isolated from chicken livers were stored in the Hubei Academy of Agricultural Sciences, Wuhan, China. ACN17 (O_78_ serotype) was used for phage isolation, and all strains were used for lytic spectrum determination. All strains were cultured overnight in Eosin Metylene Blue (EMB) (Qingdao Haibo Com Ltd., China) at 37 °C. A loopful of each strain was cultured in Luria-Bertani (LB) broth (Qingdao Haibo Com Ltd.) at 37 °C. ACN17 was resistant to gentamicin, tobramycin, trimethoprim-sulfamethoxazole, chloramphenicol by BD-Phoneix test, and stored in the Hubei Academy of Agricultural Sciences.

### 2.2 O_78_ bacteriophage isolation and purification

Intestinal contents samples of 220-day-old laying hens were collected from poultry farms in Hubei province, to isolate specific phage for the *APEC* O_78_ serotype. Samples were resuspended in SM buffer overnight at 4 °C, then centrifuged for 10 min at 10,000 × g. Each supernatant was passed through a 0.22 μm filter (Millipore, Billerica, MA, USA) to remove bacteria. The filtrate was incubated with the bacteria for the night and then centrifuged for 10 min at 10,000 × g to remove the bacteria. A total of 100 μL of each newly cultured indicator strain was mixed with 5 mL of 0.5% LB soft agar at 50 °C and then poured on the surface of a plate of prepared 1% LB agar. Detect the presence of phages using a double-layer plate, if there are transparent spots on the plate, it indicates the presence of lytic phage (Chen et al., 2020). The phage LQ5 was purified using the double-layer method and was purified five times. Purified phage LQ5 against the 10 *E. coli* indicator strains included three strains of O_1_, three strains of O_2_, and four strains of O_78_ (Table 1), as determined by the spot method (Kropinski et al., 2009). Twenty microliters of (10^8^ plaque forming units, PFU) of purified phage LQ5 was placed on a double-layer plate and the plates were incubated for 14 h at 37 °C.

**Table 1:** Lytic activity of phage LQ5 against tested strains of *APEC.*

No.	Strains of bacteria	Phage sensitivity
1	*APEC* O_1_ ACN1	−
2	*APEC* O_1_ ACN2	−
3	*APEC* O_1_ ACN3	−
4	*APEC* O_2_ ACN6	−
5	*APEC* O_2_ ACN15	−
6	*APEC* O_2_ ACN16	−
7	*APEC* O_78_ ACN17	+
8	*APEC* O_78_ ACN22	+
9	*APEC* O_78_ ACN34	+
10	*APEC* O_78_ ACN40	+

“−“indicates non-sensitivity, “+” indicates sensitivity.

### 2.3 Transmission electron microscopy (TEM) of phage LQ5

The morphology of phage LQ5 was examined by TEM (Zou et al., 2023). Purified phage LQ5 suspensions (approximately 10^9^ PFU/mL) were resuspended in 0.1 mol/L ammonium acetate and fixed on carbon-coated grids. Following staining with 2% w/v phosphotungstic acid (PTA), each grid was observed by TEM using a model HT-7700 transmission electron microscope (Hitachi High-Tech Co., Ltd., Tokyo, Japan).

### 2.4 Extraction of DNA and whole genome sequencing

The genomic DNA (gDNA) of phage LQ5 was extracted by the phenol-chloroform method (Jin et al., 2023). The phage gDNA was sequenced using the Illumina NovaSeq 2,500 platform by Shanghai Personalbio Technology Co., Ltd. Bcl2 fastq (v2.17.1.14) software was used for preliminary quality analysis to obtain raw sequencing data. We used fastp to filter and quality control the raw sequencing data obtained, cut adapters, remove low-quality reads, high-n ratio reads, and get clean reads (Chen et al., 2018). The clean reads were *de novo* assembled using the metaSPAdes software (Nurk et al., 2017). Different k-mer lengths were selected for testing and the best assembly result was obtained. Then, the clean reads were aligned to the assembled genomic sequence using the bwa software to calculate the coverage (Li and Durbin, 2009). Prediction of protein-coding genes in the genome and phage LQ5 open reading frames (ORFs) were accomplished using GeneMarkS software^1^. The protein sequence encoded by the gene was compared with the protein sequence in the database using Diamond BLASTp to infer the function of the hypothetical protein. At the same time, Bacterial and Viral Bioinformatics Resource Center (BV-BRC)^2^ was used for protein functional annotation. Based on the CARD resistance gene database^3^ was performed to predict antibiotic resistant genes (ARG) were present. VFDB data repository^4^ was performed to predict whether phage habored virulence gene. The phylogenetic tree of the terminase large subunit was constructed using the neighbor-joining method in MEGA 7 (Moon et al., 2025). The tree was drawn to scale and the units used to infer that the evolutionary distance of the phylogenetic tree were the same as the branch length. The P distance method was used to calculate the evolutionary distance. Easyfig 2.2.5^5^ was used to draw genome-wide collinearity.

### 2.5 Evaluation of the efficacy of phage LQ5 for APEC O_78_ infections in chickens

The study was approved by the Ethics Committee of the Hubei Academy of Agricultural Sciences (Approval Date: 10 March 2025; Approval Code: 5/2025). And live chickens were humanely euthanized via intravenous injection at the respective time points designated for each experimental group.

**Figure 1:**
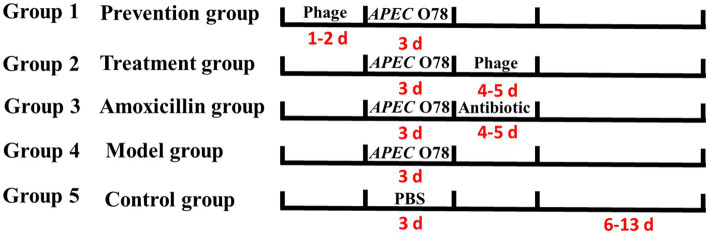
Animal experiment grouping.

To ensure 25 hatchlings, 40 specific-pathogen-free (SPF) chicken embryos were incubated in an incubator maintained at optimal temperature and humidity conditions. Subsequently, 25 healthy one-day-old chicks were transferred to isolators with *adlibitum* access to feed and water to evaluate the effect of phage LQ5 (Nicolas et al., 2023). 25 healthy one-day-old chicks were divided into five groups (*n* = 5 per group): group 1 (prevention group, administered before infection), group 2 (treatment group, administered after infection), group 3 (amoxicillin group), group 4 (model group), and group 5 (control group). In the first day, 5 chicks in group 1 (prevention group) were orally administered phage LQ5. Groups of 2, 3, and 4 treated wih phage LQ5, amoxicillin, and phosphate buffered saline (PBS), respectively. In the third day, groups of 1, 2, 3, and 4 were challenged intratracheally with 10^8^ colony forming units (CFU) of *APEC* O_78_. The group 5 were as negative control, with only PBS being administered. In the 4th and 5th days, groups 2 and 3 were orally administered with phage and amoxicillin, respectively. The medication used during the amoxicillin group was commercial amoxicillin, with the veterinary drug code 120191199. It was used in accordance with the instructions. The concentration of the phage LQ5 was 10^9^ PFU/mL. Livers and spleens from the five groups were aseptically removed at 10 days post-infection (dpi) to determine the load of *APEC* O_78_ (Galal et al., 2021). Briefly, 1 g of liver and spleen of each group were suspended in PBS and homogenized with an Omnimixer homogenizer. Tissue homogenates were serially diluted 10-fold in PBS, and 100 μL of each dilution was plated onto MacConkey-lactose agar plates for bacterial enumeration. The plates were incubated at 37 °C for 24 h, after which the number of pink colonies was counted and expressed as CFU per gram of tissue. Hematoxylin and eosin (H&E) staining was used to assess the injury in different tissues of five groups. The description of organizational changes was based on the book “Pathological Basis of Veterinary Diseases (5th Edition)”, ISBN: 978-7-109-20098-2 (Figure 1).

### 2.6 Statistical analysis

One-way analysis of variance (ANOVA) was used to evaluate the difference between the experimental and control groups. GraphPad Prism 8.0 (GraphPad Software, San Diego, CA, USA) was used toplot the data. *p* < 0.05 was considered statistically significant.

## 3 Results

### 3.1 Isolation, morphology and host range of phage LQ5

**Figure 2:**
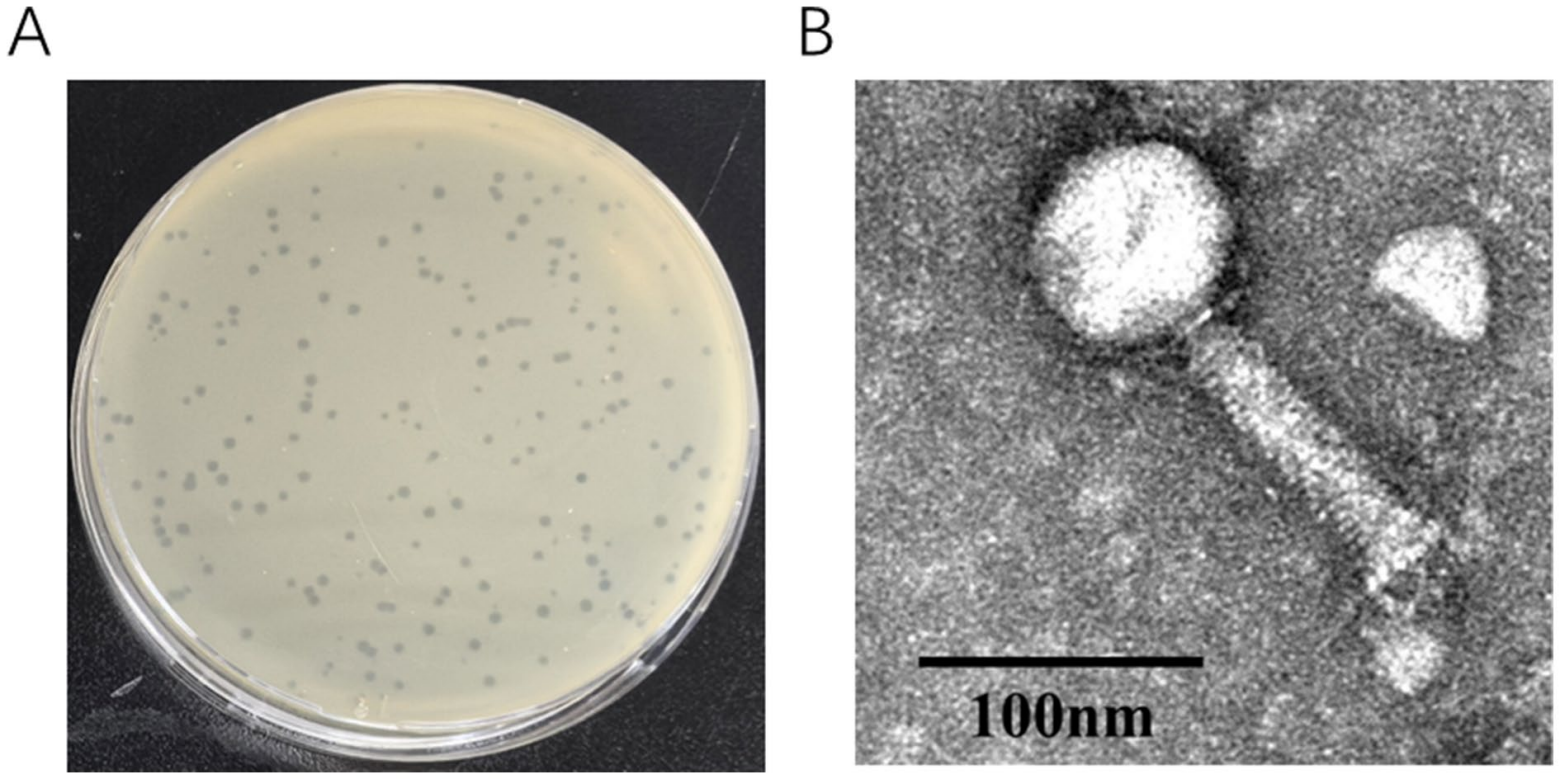
**(A)** Isolation and purification of O_78_ phage LQ5. **(B)** Transmission electron micrograph of phage LQ5. The scale is 50 nm.

Phage LQ5 was isolated from the intestinal contents of laying hens using the double-layer agar method, with *APEC* O_78_ ACN17 strains serving as the host. The purified phage LQ5 formed several transparent plaques on the plate, with approximate diameters of 2 mm (Figure 2A). TEM revealed that phage LQ5 belongs to the *Myoviridae* family (Figure 2B). The lytic activity of LQ5 to 10 *APEC* strains is shown in Table 1. LQ5 exhibited lytic activity exclusively against O_78_ *APEC* serotypes and showed no infectivity toward O_1_ or O_2_ serotypes.

### 3.2 Genome analysis of phage LQ5

**Figure 3:**
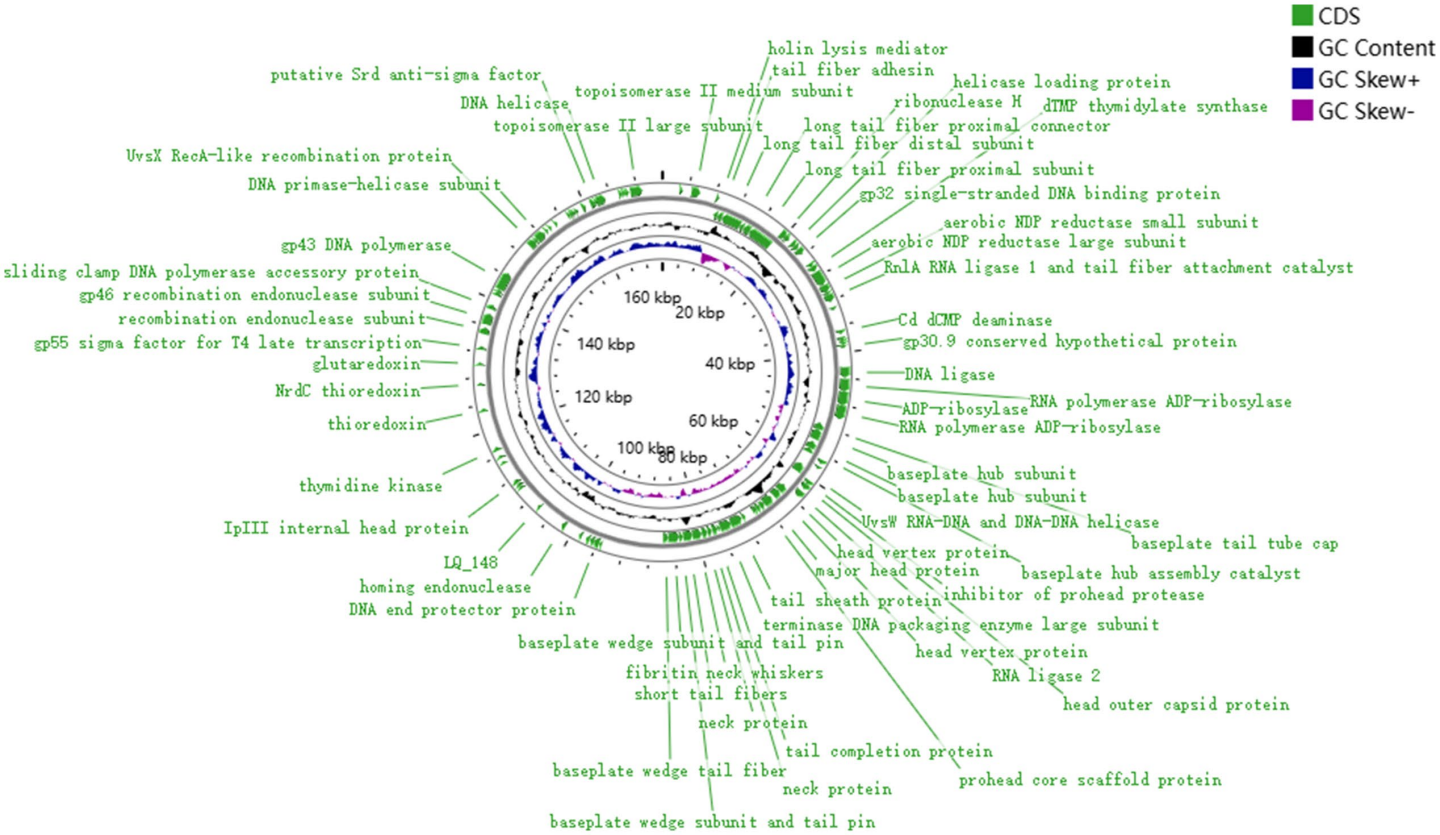
Genome map of phage LQ5 generated by CGView. The regions in green represent the distribution of the coding sequence (CDS) region and the arrows indicate the direction of transcription. The total GC content (39.52%) is indicated in black, while the inner ring with blue and purple histograms indicates GC skew. For clarity, the hypothetical protein is not described on the map.

**Figure 4:**
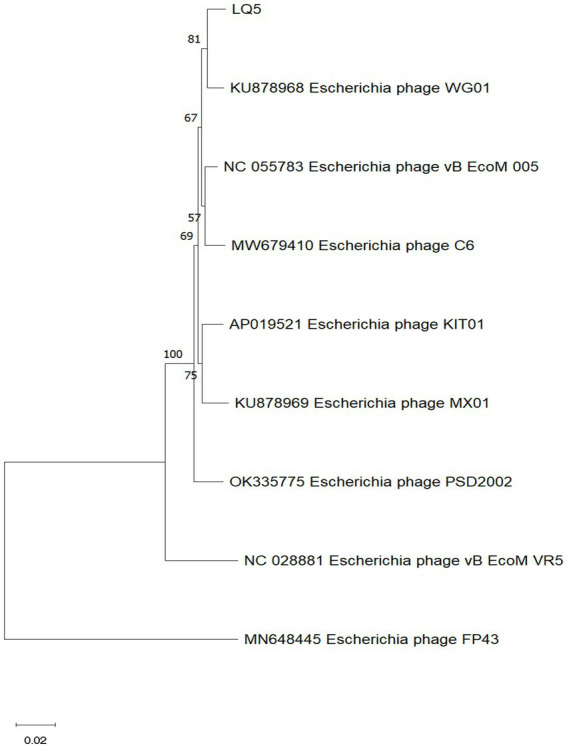
Cluster analysis of *APEC* O_78_ phage LQ5. The phylogenetic tree was constructed based on the neighbor-joining method of the terminase large subunit.

**Figure 5:**
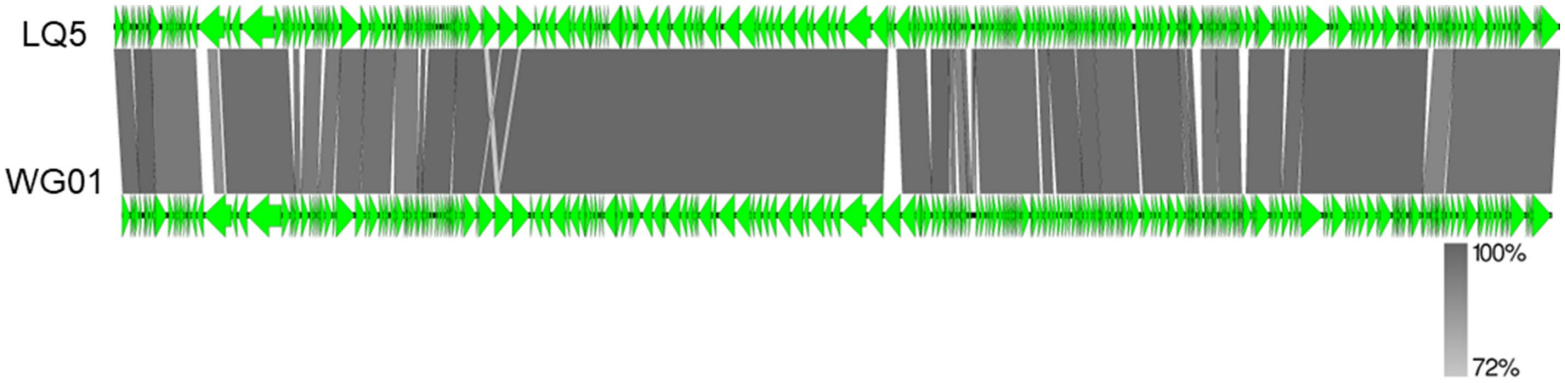
Genome homology analysis of phage LQ5 with WG01. The green arrow represents the open reading frames. The color gradient represents the level of nucleotide identity between the phage genomes.

The gene map of phage LQ5 was shown in Figure 3. Phage LQ5 is a double-stranded DNA phage with a genome length of 171,908 bp and a GC content of 39.52% (GenBank: OR677401). The genome sequence of LQ5 was compared online by BLASTN. The phage with the highest genome homology (98.60%) was *E. coli* phage WG01 (GenBank: KU878968.1). Multiple genome alignments and phylogenetic tree analyses of phage LQ5 and *E. coli* phage WG01, 005, C6, KIT01, MX01, PSD2002, VR5, and FP43 are shown in Figure 4. Easyfig 2.2.5 (see text footnote 5) was used to map the genome-wide collinearity ratio. The collinearity of phages WG01 and LQ5 was extremely high (Figure 5). Phage WG01 was isolated from Nanjing, Jiangsu Province, China. Some genes were located in different positions on the DNA chain, which might be a phenomenon of gene rearrangement that occurs in phages in order to enhance diversity and adaptability.

The results of protein functional annotation using BV-BRC were consistent with those of GeneMarkS. Protein analyses of phage LQ5 are presented in Table 2. The clarified function of the proteins provide a theoretical basis for subsequent studies of functional proteins of phages. The phage genome consists of four known gene cluster modules. DNA replication and modification modules include DNA helicase (ORF261) and DNA polymerase (ORF227). The DNA packaging module includes terminase large subunit (ORF108) and terminase small subunit (ORF109). The lysis module includes lysozyme (ORF158) and holin (ORF23). Finally, structural proteins include tail fiber protein (ORF62), baseplate protein (ORF117), capsid protein (ORF94) and scaffold protein (ORF101). ORF100 encodes the phage principal prtein. ORF130 encodes the main tail fiber assembly protein of phage, while ORF62 encodes the tail fiber of phage. Phage LQ5 has a binary lysis system composed of holin and lysozyme, which can specifically destroy the cell membrane and cell wall of bacteria, respectively, with a completely different mechanism of action from antibiotics (Washizaki et al., 2016). Glutaredoxin (ORF202) was annotated in LQ5. The phylogenetic tree of LQ5 was constructed based on the nucleotide sequence of the terminase large subunit (ORF108). The tail fiber protein was responsible for the specific initial recognition of host bacteria and can be a potential biological cognitive element for detecting bacteria (Wintachai et al., 2024). No phage, transposase, excision enzyme homology, and repressor was predicted in the LQ5 genome. According to the above sequencing results, phage LQ5 was considered a novel phage.

**Table 2:** Analysis of main proteins of phage LQ5.

ORF	Start	Stop	Strand	Function
LQ5_7	2,763	2,873	+	inner membrane protein
LQ5_13	4,365	5,693	+	topoisomerase II medium subunit
LQ5_22	8,285	8,560	+	anti-sigma 70 protein
LQ5_23	8,557	9,210	−	holin lysis mediator
LQ5_24	9,252	10,052	−	tail fiber adhesin
LQ5_25	10,086	13,307	−	long tail fiber distal subunit
LQ5_26	13,316	13,963	−	large distal long tail fiber subunit
LQ5_27	14,039	15,160	−	long tail fiber proximal connector
LQ5_28	15,169	19,218	−	long tail fiber proximal subunit
LQ5_29	19,326	20,273	+	ribonuclease H
LQ5_30	20,284	20,553	+	dsDNA binding protein
LQ5_31	20,534	20,836	+	gp33 late promoter transcription accessory protein
LQ5_33	21,512	22,165	+	helicase loading protein
LQ5_34	22,153	22,782	+	homing endonuclease
LQ5_35	22,887	23,792	+	gp32 single-stranded DNA binding protein
LQ5_41	25,603	26,184	+	dihydrofolate reductase
LQ5_42	26,181	27,038	+	dTMP thymidylate synthase
LQ5_43	27,167	29,422	+	aerobic NDP reductase large subunit
LQ5_44	29,476	30,615	+	aerobic NDP reductase small subunit
LQ5_45	30,619	31,056	+	endonuclease II
LQ5_46	31,061	32,209	+	RnlA RNA ligase 1 and tail fiber attachment catalyst
LQ5_49	33,105	33,395	+	conserved hypothetical predicted membrane protein
LQ5_59	36,642	37,187	+	Cd dCMP deaminase
LQ5_62	37,752	38,051	+	tail fibers protein
LQ5_63	38,110	38,433	+	head assembly cochaperone with GroEL
LQ5_64	38,580	38,828	+	rIII lysis inhibition accessory protein rapidlysis phenotype
LQ5_65	39,091	39,252	+	gp30.9 conserved hypothetical protein
LQ5_74	42,259	43,782	+	DNA ligase
LQ5_76	44,017	45,912	+	RNA polymerase ADP-ribosylase
LQ5_77	45,921	48,002	+	ADP-ribosylase
LQ5_78	48,006	50,129	+	RNA polymerase ADP-ribosylase
LQ5_81	51,465	52,553	−	baseplate tail tube cap
LQ5_82	52,562	54,298	−	baseplate hub subunit
LQ5_85	55,929	56,681	−	baseplate hub assembly catalyst
LQ5_88	57,803	58,237	+	UvsY recombination repair and ssDNA binding protein
LQ5_92	58,818	60,314	−	UvsW RNA–DNA and DNA–DNA helicase
LQ5_93	60,370	61,041	+	inhibitor of prohead protease
LQ5_94	61,051	61,899	+	head outer capsid protein
LQ5_96	62,327	63,334	+	RNA ligase 2
LQ5_97	63,361	64,641	−	head vertex protein
LQ5_98	64,641	65,924	−	head vertex protein
LQ5_100	66,342	67,901	−	major head protein
LQ5_101	67,923	68,738	−	prohead core scaffold protein
LQ5_102	68,769	69,404	−	prohead core scaffold protein and protease
LQ5_103	69,404	69,832	−	prohead core protein
LQ5_104	69,832	70,077	−	prohead core protein precursor
LQ5_106	71,709	72,200	−	tail tube protein
LQ5_107	72,386	74,368	−	tail sheath protein
LQ5_108	74,401	76,236	−	terminase large subunit
LQ5_109	76,220	76,711	−	terminase small subunit
LQ5_110	76,708	77,574	−	tail completion protein
LQ5_111	77,614	78,387	−	neck protein
LQ5_112	78,390	79,325	−	neck protein
LQ5_113	79,380	80,969	−	fibritin neck whiskers
LQ5_114	80,979	82,505	−	short tail fibers
LQ5_115	82,505	83,170	−	baseplate wedge subunit and tail pin
LQ5_116	83,170	84,981	−	baseplate wedge subunit and tail pin
LQ5_117	84,981	85,841	−	baseplate wedge tail fiber
LQ5_125	95,206	95,655	+	head completion protein
LQ5_126	95,652	96,479	+	DNA end protector protein
LQ5_127	96,479	97,129	+	putative site-specific intron-like DNA endonuclease
LQ5_128	97,212	97,802	+	tail completion and sheath stabilizer protein
LQ5_130	98,556	98,801	+	tail fiber assembly protein
LQ5_138	101,288	101,953	+	homing endonuclease
LQ5_157	110,022	110,471	+	nudix hydrolase
LQ5_158	110,502	110,990	+	lysozyme murein hydrolase
LQ5_159	111,078	111,674	+	IpIII internal head protein
LQ5_168	114,567	115,025	+	site-specific RNA endonuclease
LQ5_170	115,578	115,934	+	valyl-tRNA synthetase modifier
LQ5_174	116,762	117,334	+	thymidine kinase
LQ5_186	122,955	123,479	+	thioredoxin
LQ5_193	126,985	127,248	+	NrdC thioredoxin
LQ5_202	130,012	130,278	+	glutaredoxin
LQ5_210	132,275	132,832	+	gp55 sigma factor for T4 late transcription
LQ5_216	134,667	135,689	+	recombination endonuclease subunit
LQ5_219	136,145	137,827	+	gp46 recombination endonuclease subunit
LQ5_222	138,516	139,214	+	sliding clamp DNA polymerase accessory protein
LQ5_225	140,741	141,313	+	clamp loader subunit DNA polymerase
LQ5_226	141,320	141,682	+	translational repressor protein
LQ5_227	141,771	144,482	+	DNA polymerase
LQ5_235	149,712	150,884	+	UvsX RecA-like recombination protein
LQ5_236	150,877	151,206	+	gp40 head vertex assembly chaperone
LQ5_237	151,200	152,630	+	DNA primase-helicase subunit
LQ5_239	153,032	153,226	+	discriminator of mRNA degradation
LQ5_242	153,667	153,960	+	sp. spackle periplasmic protein
LQ5_245	154,799	154,960	+	gp61.1 hypothetical protein
LQ5_249	156,948	157,469	+	gp56 dCTPase
LQ5_250	157,511	157,744	+	small outer capsid protein
LQ5_251	157,839	158,048	+	Mrh.2 hypothetical protein
LQ5_253	158,362	158,562	+	transcription modulator
LQ5_257	159,448	160,017	+	ModB ADP-ribosylase
LQ5_258	160,072	160,686	+	ADP-ribosylase
LQ5_259	160,825	161,574	+	putative Srd anti-sigma factor
LQ5_260	161,579	161,875	+	hypothetical protein AVT32_gp016
LQ5_261	161,872	163,203	+	DNA helicase
LQ5_266	165,310	165,861	+	modifier of transcription
LQ5_267	165,934	166,152	+	modifier of suppressor tRNAs
LQ5_268	166,145	166,549	+	putative RNA metabolism modulator
LQ5_269	166,551	166,727	+	gp39.2 hypothetical protein
LQ5_271	167,061	168,878	+	topoisomerase II large subunit

### 3.3 Efficacy of phage LQ5 concerning bacterial load in chickens and survival

#### 3.3.1 The survival rate of chickens

**Figure 6:**
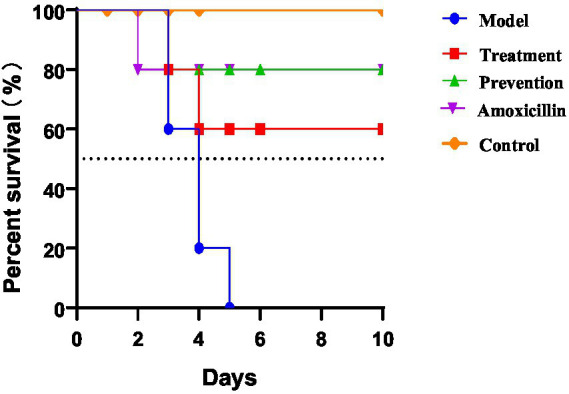
Survival rates between different groups.

We used the one-day SPF chicken model to study the effect of phage on *APEC* O_78_ ACN17 infection in chickens. The five groups were compared concerning the content of *E. coli* O_78_ in liver and spleen. The groups of model, treatment, prevention, and amoxicillin displayed survival rates of 0, 60, 80, and 80%, respectively (Figure 6).

Survival of the different treatment groups was assessed at 10 days post-infection (10 dpi). The model group, challenged intratracheally with 10^8^ CFU of *APEC* O_78._ The control group was treated with an equivalent dose of PBS; The prevention group received phage prophylaxis 24 h prior to bacterial challenge, whereas the treatment group received phage therapy at 1 day post-infection (1dpi), following an intratracheal challenge with 10^8^ CFU of *APEC* O_78_. The amoxicillin group was received amoxicillin therapy at 1 day post-infection (1dpi). The control group maintained a 100% survival rate throughout the observation period (days 0–10). The prevention group and the amoxicillin group had a survival rate of around 80%. The treatment group maintained a survival rate of around 60%. The model group experienced a sharp decline in survival rate after day 4, with a survival rate of approximately 0 around day 5.

#### 3.3.2 The bacterial load in the liver and spleen

**Figure 7:**
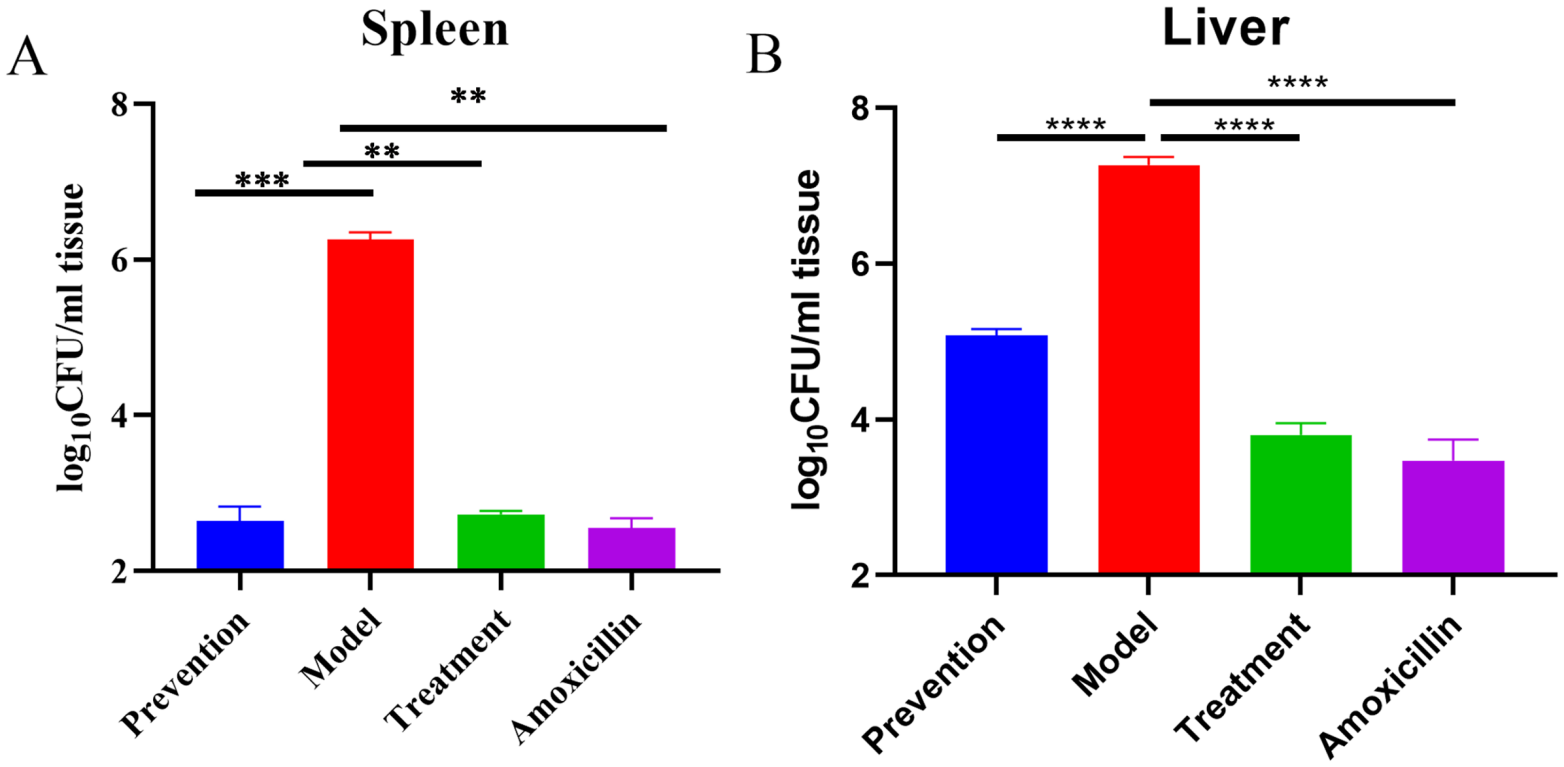
Bacterial counts of liver and spleen. **(A)** The number of bacteria in the spleen. **(B)** The number of bacteria in the liver.

We used the bacterial loads in the liver and spleen to evaluate the efficacy of the different treatment groups. Compared with the model group, the bacteria loads in spleens in the groups of treatment, prevention, and amoxicillin have significant difference, especially in liver (*p* < 0.0001) (Figure 7). The results show that the bacterial load in the spleen of the model group is significantly higher than that of the prevention group (***p < 0.0001), treatment group (**p < 0.001), and amoxicillin group (**p < 0.001). There is no significant difference among the prevention group, treatment group, and amoxicillin group (Figure 7A). The results show that the bacterial load in the liver of the model group is significantly higher than that of the prevention group (***p < 0.0001), treatment group (***p < 0.0001), and amoxicillin group (***p < 0.0001). The bacterial load in the prevention group is higher than that in the treatment group and amoxicillin group, and there is no significant difference between the treatment group and amoxicillin group (Figure 7B).

The x-axis represents different groups (prevention, model, treatment, amoxicillin), and the y-axis represents (CFU/mL tissue). All samples were collected at 7 dpi (7 days post-infection), and the data are represented by the mean (CFU/mL) of samples from each group. *p < 0.05, **p < 0.01, ***p < 0.001, ****p < 0.0001.

#### 3.3.3 Morphological observation of the different tissue

**Figure 8:**
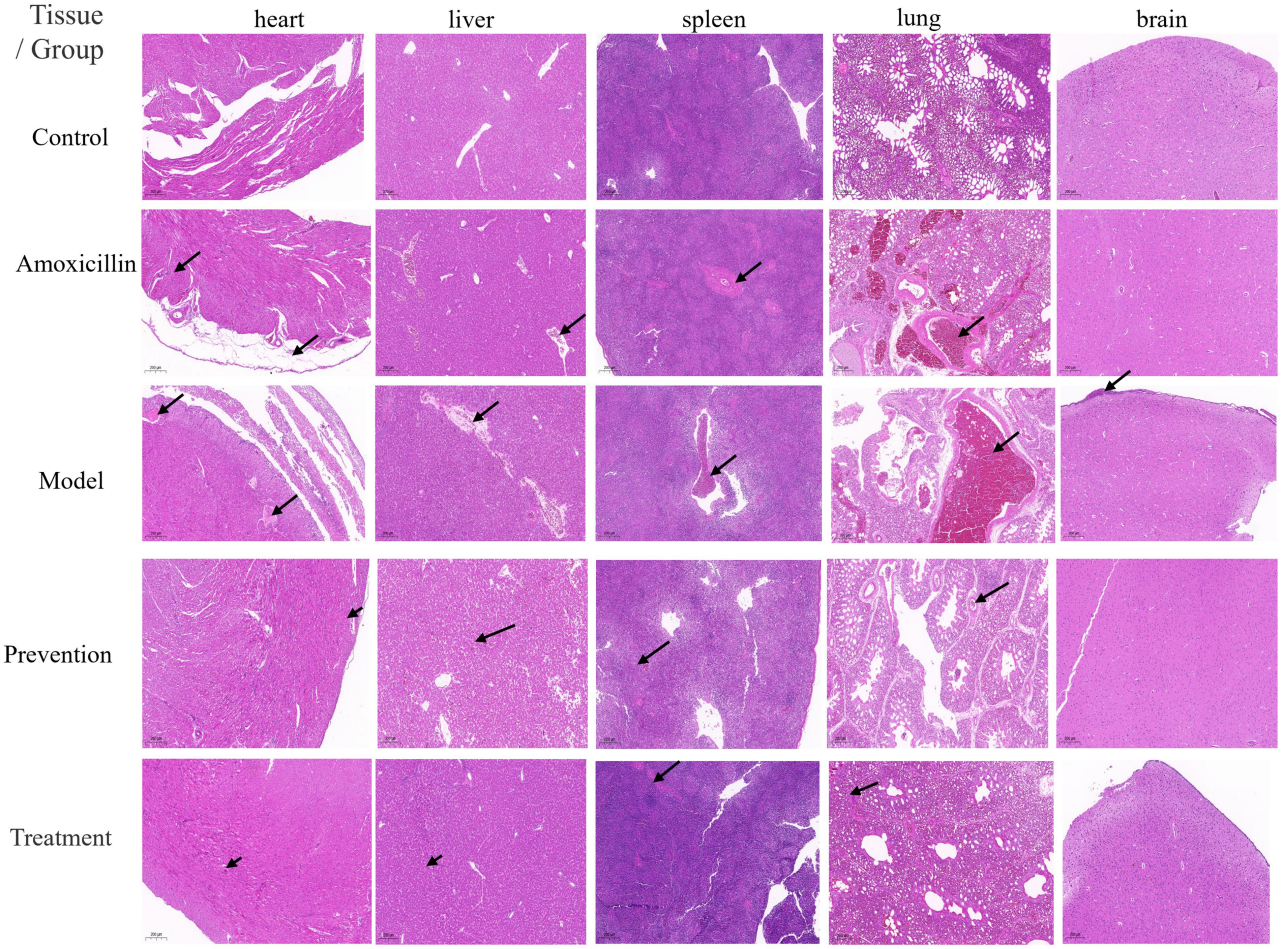
Histology of chicken tissue sections after H&E staining at 200 × magnification. Histology of chicken tissue sections (heart, liver, spleen, lung, and brain) was observed after H&E staining at 200 × magnification.

Histology analysis revealed some traits in the five tissue types. In the model group, many sites of bleeding were evident in liver sinusoids and cerebral glial membrane; with many red blood cells in the medulla of the spleen, the alveoli of the lungs (along with a widening of the alveolar space), the lungs, the liver. No obvious pathological changes were observed in the other groups. Compared with the model group, the preventive and treatment groups displayed only a small amount of bleeding, while the antibiotic group had more severe symptoms than the preventive and treatment groups. Antibiotics and phages have similar effects in reducing the bacterial load in the liver and spleen. Pathological section results showed that antibiotics and phages have similar effects in reducing the bacterial load in the liver and spleen, and phages have a better protective effect on the liver and spleen tissues (Figure 8).

The groups include control, amoxicillin, model, prevention, and treatment, with arrows indicating lesions. The control group showed no obvious lesions, with normal tissue structures. Lesions (such as inflammation and tissue damage, indicated by arrows) were observed in the heart, liver, spleen, and lung tissues of the amoxicillin group, model group, and prevention group. The model group had relatively more obvious lesions, while the amoxicillin group and prevention group had lesions to varying degrees. The Treatment group had few lesions in each tissue, close to normal. Interestingly, among all the groups, only the Model group showed vascular cuffing lesion in the brain tissue.

## 4 Discussion

Phages specifically target pathogenic bacteria without harming beneficial microorganisms (Zhao et al., 2025; Xu et al., 2025). The primary pathogens responsible for intestinal diseases in animals are *E. coli*, *Salmonella*, and *Clostridium perfringens*, with colitis being the most frequent manifestation (Hegarty, 2025). A significant challenge in prevention and treatment is the widespread resistance to first-line antibiotics, necessitating the identification of suitable alternatives such as phage therapy (Antimicrobial Resistance Collaborators, 2022).

Phage LQ5 and vB_EcoM_*APEC* both belong to the *Myoviridae* family and are sensitive to *APEC* O_78_ (Deng et al., 2021). The characteristics of phage such as MOI, pH tolerance and temperature stability will be reflected in another article about the mechanism of phage resistance. Functional annotation of phage genomes and analysis of their roles in the phage life cycle are essential to determine aspects such as phage interaction, replication, infection of bacteria, coevolution, and host range (Tokodi et al., 2025; Doekes et al., 2021; Holtappels et al., 2023). Phage tail fiber protein can be modified by natural evolution or genetic engineering, including homologous recombination, synthetic biology and directed evolution, to expand their host range or improve their specific recognition ability (Filik-Matyjaszczyk et al., 2025).

The improper use of antibacterial drugs in poultry farming is one of the reasons for the emergence of antimicrobial resistance (AMR) in poultry production. Antibiotic resistant bacteria caused a 1.27 million human deaths in 2019 globally (Iskender and Soyer, 2023). These strategies include phages (Mao et al., 2023) herbal medicines (Zhang et al., 2020), probiotics (Zhang et al., 2023), synthetic CpG oligodeoxynucleotide (Gunawardana et al., 2022). Phages have a positive preventive and therapeutic effect in combating and preventing the drug resistance of *APEC* strains. Phages reduced the mortality rate of one-day-old chickens by 20 and 30%, respectively, (in the treatment group and the prevention group) (Jhandai et al., 2024). Other studies reported significantly reduced growth of two kinds of *APEC* treated with phage compared to a control group not treated with phage. PEC9 was a siphovirus-like phage, which lysed 9/20 O_1_ and 6/20 O_2_ serotypes of *E. coli* (Lau et al., 2010). These findings implicate PEC9 as having an important role in increasing survival rate, and reducing bacterial loads and liver lesions in *APEC* infections. Researchers identified vB_EcoM as a member of *Myoviridae*, with lytic activity for the *APEC* O_78_ sereotype (Balcao et al., 2022). In contrast, purified endolysin displayed broad-spectrum lytic activity for the O_78_, O_157_ sereotypes of *APEC*, as well as *Klebsiella, Salmonella*, *Shigella*, and *Yersinia* strains (Yao et al., 2023). These findings highlight the importance of evaluating phage with amoxicillin concerning bacteria loads. The preventive and therapeutic results of phage LQ5 are similar to those of antibiotics, providing a reference for the clinical application of phages and offering an alternative to antibiotics for the prevention and control of *E. coli* O78 infection.

Infection rates ranged from 20 to 85.7% when each phage was used alone and from 78.6 to 88.9% when antibiotics were used (El-Meihy et al., 2024). The mice that received phage therapy did not develop pneumonia caused by multi-drug resistant *Staphylococcus aureus*. This can be used as a basis for exploring the application of phage therapy in treating *Staphylococcus aureus* infections in blood (Oduor et al., 2016). In the model of *Pseudomonas aeruginosa* sinusitis, the safety and effectiveness of phage cocktail *in vivo* provide a basis for the application of phage (Fong et al., 2019). Studies have shown that in the first stage under bioimaging, the liver and spleen can observe the most obvious bacterial distribution, and tissue lesions in the lungs are more typical, providing reference for our subsequent assessment of bacterial load, thus making the lungs more convincing as an important organ and as a progressive regulator of colisepticaemia (Abdelhamid et al., 2024). The application of these phages has indeed greatly enriched the knowledge and practice of phage safety.

In this study, we successfully isolated a novel *APEC* phage LQ5, which can specifically lyse the O_78_ serotype of *E. coli*. Phage LQ5 reduced the *APEC* counts in the liver and spleen of chickens, indicating protective effects of chickens infected with *APEC* O_78_. Importantly, these results are consistent with previous reports (Deng et al., 2021; Moreno et al., 2025). Phage LQ5 exhibited antibacterial activity comparable to amoxicillin against the O_78_ serotype. These results further support the potential of phages to reduce antibiotic use in poultry production.

## 5 Conclusion

This study evaluated the efficacy of phage LQ5 against *E. coli* O_78_ infection in chicks. Phage LQ5 increased the survival rate of chicks infected with *E. coli*, reduced the bacterial load in the spleen and liver, and alleviated the pathological changes in tissues. This study provides a theoretical basis for the use of phages in the prevention of *E. coli* in chicks, and offers a strategy for the clinical prevention and treatment of colibacillosis. The limitations of this study are the insufficient research on the structure and function of the phage receptor binding protein and the unclear mechanism of host recognition, which will be carried out in future research.

## Data Availability

The data presented in this study are publicly available. The data can be found at: https://www.ncbi.nlm.nih.gov/nuccore/OR677401.

## Footnotes

1
http://topaz.gatech.edu/Genemark/

2
https://www.bv-brc.org/

3
https://card.mcmaster.ca/

4
http://www.mgc.ac.cn/VFs/

5
http://mjsull.github.io/Easyfig/
